# Acute Encephalopathy in a 10-Year-Old Patient With Maple Syrup Urine Disease: A Challenging Diagnosis

**DOI:** 10.7759/cureus.53043

**Published:** 2024-01-27

**Authors:** Pedro Miragaia, Ana Grangeia, Esmeralda Rodrigues, Raquel Sousa, Augusto Ribeiro

**Affiliations:** 1 Department of Pediatrics, Centro Hospitalar Universitário de São João, Porto, PRT; 2 Department of Genetics, Centro Hospitalar Universitário de São João, Porto, PRT; 3 Faculty of Medicine, University of Porto, Porto, PRT; 4 Inborn Errors of Metabolism Unit, Reference Center for Inherited Metabolic Diseases, Centro Hospitalar Universitário de São João, Porto, PRT

**Keywords:** pediatrics neurology, neurology and critical care, acute metabolic encephalopathy, paediatric intensive care unit, metabolic encephalopathy

## Abstract

Maple syrup urine disease (MSUD) is a rare autosomal recessive metabolic disorder characterized by a deficiency in the branched-chain alpha-keto acid dehydrogenase complex, leading to the toxic accumulation of leucine, isoleucine and valine. Acute encephalopathy (AE) is a severe neurological disorder with diverse etiologies, demanding prompt identification and intervention.

We present a unique case of a previously healthy teenage patient who developed AE during an influenza infection. Despite initial inconclusive investigations, the patient's condition rapidly deteriorated, requiring pediatric intensive care unit (PICU) admission. Diagnostic challenges included fluctuating mental status and refractory intracranial hypertension, ultimately necessitating decompressive craniectomy. Empirical treatments, including corticosteroids, tocilizumab, and plasmapheresis, were administered. Finally, clinical exome analysis revealed a pathogenic variant in homozygosity in the BCKDHA gene associated with MSUD type Ia. Her adult sister, experiencing similar symptoms in the same time period, did not survive.

This case underscores the importance of considering metabolic disorders in AE etiology, even accounting for its various associated syndromes and usual prolonged diagnostic investigation, as prompt treatment initiation is vital for improved outcomes. Management of AE involves addressing seizures, systemic support and neuromonitoring, namely, intracranial pressure monitoring. Inborn errors of metabolism, like MSUD, should be considered, even if universally screened, as delayed diagnosis can result in prolonged hospitalization and significant morbidity.

## Introduction

Maple syrup urine disease (MSUD), also referred to as leucinosis, is a rare autosomal recessive metabolic disorder characterized by a deficiency in the branched-chain alpha-keto acid dehydrogenase complex [1]. It occurs in approximately in 1/185,000 live births globally. This enzymatic deficiency leads to the toxic accumulation of leucine, isoleucine and valine, resulting in a spectrum of clinical manifestations. Although MSUD is renowned for its impact on the neurological and metabolic systems, particularly in newborns, occurrences of necrotizing encephalopathy linked to this condition, especially in adolescents, are exceedingly infrequent [2].

Clinically, MSUD may manifest in five different phenotypes: classic, intermittent, intermediate, thiamine-responsive, and E3 deficient. These phenotypes are classified based on specific variants, residual enzyme activity, the specific age of onset or presentation, response to particular treatments, and the severity of clinical symptoms [3].

Acute encephalopathy (AE) is a severe and rapid-onset neurological disorder characterized by a generalized dysfunction of the brain. This condition is often marked by altered mental status, including confusion, seizures, and in some cases, coma. Acute encephalopathy can be caused by various factors, such as infections, metabolic disturbances, toxins, seizures or cytokine storm [4]. The rapid progression of symptoms demands prompt medical attention to identify and address the underlying cause. Early intervention is crucial to prevent further neurological damage and improve the prognosis [5].

We hereby present one rare case of a teenage patient with acute encephalopathy as the initial presentation and MSUD (intermediate type). We aim to outline the clinical presentation, diagnostic challenges, treatment strategies, and disease course in our patient, emphasizing the importance of increased awareness and early intervention.

## Case presentation

A 10-year-old female, second child of a non-related couple, previously healthy and residing in a rural area of Portugal, presented to the Emergency Department with behavior changes, visual hallucinations, and temporal and spatial disorientation. Over the preceding four days, she experienced fever (during the first two days), myalgia, mild cough, and rhinorrhea, suspecting of a viral illness. She received only supportive measures and did not take any specific medicines or alternative therapies for cough. There was no history of any trauma. Her 18-year-old sister showed similar respiratory symptoms and was also taken to the Emergency Department that day due to seizures and altered mental state. She was later admitted to an adult neurological intensive care unit.

The initial evaluation in the physical examination revealed no alterations, except for progressively worsening impaired mental status, demanding transfer to a hospital center with a pediatric intensive care unit (PICU). Laboratory analysis showed no serum electrolyte or glucose alterations and a slight metabolic acidosis (pH 7.22, pCO_2_ 44 mmHg, HCO3^-^ 18 mmol/L) with an increased anion gap (20 mEq/L). The virologic panel of the nasopharyngeal swab was positive only for influenza A (polymerase chain reaction). The C-reactive protein result was negative (<0.5 mg/L), with a normal white cell count, and the screening for urine toxics was negative as well. Liver transaminases and coagulation studies were within normal ranges (NRs). Cerebrospinal fluid (CSF) analysis showed no alterations in cytologic, bacteriological or virological examinations.

Upon arrival at our center, she exhibited a fluctuating mental status (Glasgow Coma Scale, or GCS, score from 10 to 15), with periods of bradycardia, leading to hospitalization in the PICU under the provisional diagnosis of acute encephalopathy due to influenza A. A brain computerized tomography (CT) scan revealed no alterations. Empiric therapy was initiated with ceftriaxone, oseltamivir, and acyclovir.

During day 1 in the PICU, she experienced rapid clinical worsening with establishment of seizures and impaired mental status in the postictal period (GCS score 8), prompting endotracheal intubation and starting sedoanalgesia with midazolam and fentanyl. A repeated CT scan showed no alterations. Blood gas analysis at this point revealed no disturbances in the acid-base equilibrium or metabolic alterations. Electroencephalogram (EEG) showed a pattern of severe diffuse cerebral dysfunction, particularly in the right fronto-temporal area, without epileptiform activity. An intracranial pressure (ICP) catheter was introduced that day to monitor ICP.

**Figure 1:**
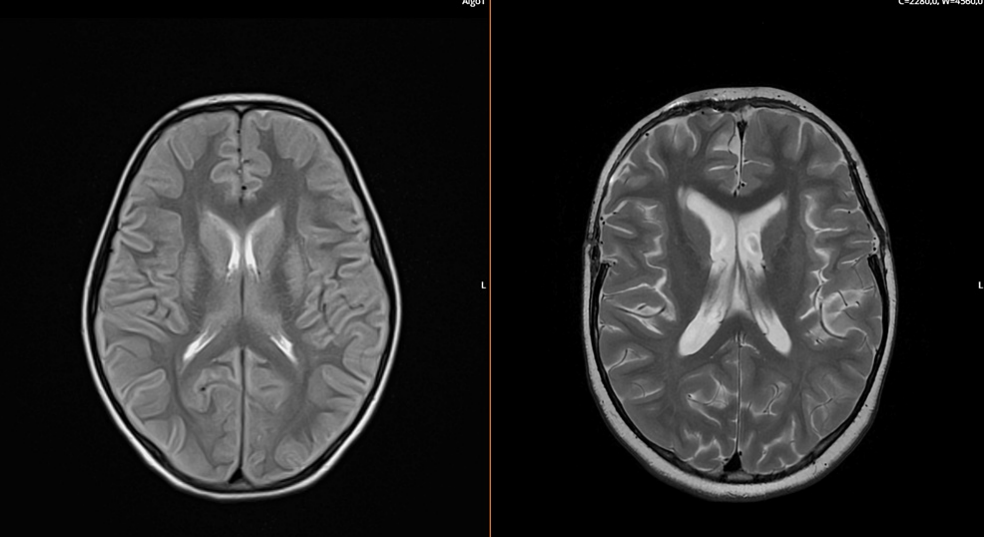
A comparison of the magnetic resonance images on the fourth day of hospitalization (left) and after recovery (right). Note the cytotoxic edema associated with a swollen appearance, resulting in a reduction in the patency of cortical sulci

Treatment at this point included endovenous methylprednisolone in pulses of 30 mg/kg for five days, intravenous immunoglobulin (IvIg), and plasmapheresis. A repeated brain image on day 4, with magnetic resonance (Figure 1), showed diffuse cytotoxic edema, and she presented a progressive increase in ICP, difficult to control with osmotic therapy. Empirically, a single dose of tocilizumab was administered.

On day 5 of hospitalization, after an episode of anisocoria, with continuing refractory intracranial hypertension (ICH; maximum ICP 70 mmHg), she underwent brain CT again that revealed imagiological impairment, with diffuse brain edema and loss of white-grey matter differentiation. Given this scenario, she underwent decompressive craniectomy. Yet, she maintained ICH with very poor control until day 12, even with all the measures, requiring noradrenaline support (from days 6 to 14) to provide adequate brain perfusion.

She had a prolonged hospitalization period of 59 days (32 in the PICU), receiving treatment with plasmapheresis for five days, thiamine supplementation, repeated treatment with IvIg, and tocilizumab. Sedoanalgesia weaning began on day 19, coinciding with progressive neurological recovery. An infectious disease study with serological, cultural, and molecular biology studies was entirely negative, except for influenza A in the nasopharyngeal swab. Cytokine analysis showed no alterations.

Afterwards, she underwent an extended hospitalization at a physical rehabilitation center, ultimately being discharged seven months after the initial presentation. Currently, approximately 10 months post-episode, she exhibits only facial peripheral palsy and mild dysarthrophonia (voice and speech impairment). Her sister died on day 4 of hospitalization, after rapidly progressive coma, with refractory cranial hypertension.

Trio exome sequencing revealed a pathogenic variant c.659C>T (p.(Ala220Val)) in homozygosity in the BCKDHA (NM_000709.4) gene establishing the diagnosis of MSUD. Genetic screening of the identified BCKDHA variant in stored DNA from the deceased sister identified the same genotype, also confirming the diagnosis of MSUD in her sister.

At this time, when she was already asymptomatic, plasma amino acids revealed elevations in branched-chain amino acids (BCAAs) and alloisoleucine, confirming an intermediate form of MSUD (valine, 399 μmol/L, NR 88-290 μmol/L; leucine, 273 μmol/L, NR 68-158 μmol/L; isoleucine, 128 μmol/L, NR 36-82 μmol/L; alloisoleucine, 17 μmol/L, NR 1.2-3.4 μmol/L). She was started on a mild protein-restricted diet supplemented with specific BCAA-free amino acid mixture as chronic treatment and an emergency protocol for intercurrent acute illness to avoid the risk of metabolic decompensation. While asymptomatic she had persistent elevations of alloisoleucine (a range of 8-11 μmol/L).

## Discussion

AE manifests as syndromes characterized by a sudden, prolonged impairment of consciousness. Typically affecting previously healthy children, AE may result from various factors, including infections (notably influenza virus, and other microorganisms such as herpesvirus, varicella-zoster, or mycoplasma), convulsive status epilepticus, cytokine storm (as in acute necrotizing encephalopathy), and metabolic, endocrine, or electrolytic disorders (e.g., Reye syndrome or congenital adrenal hyperplasia) [4]. The clinical presentation may be subtle initially, emphasizing the need for heightened awareness among physicians due to its life-threatening potential and possible long-lasting sequelae [5].

In the present case, a previously healthy teenage girl developed AE symptoms during an influenza infection. Additionally, her 18-year-old sister displayed similar symptoms concurrently, prompting concerns about a genetic disorder or toxic etiology. The importance of identifying the underlying cause of AE is underscored, as it has therapeutic implications. In fact, as infectious diseases such as influenza may be directly responsible for the clinical syndrome [4], with direct cytotoxic activity by the virus, it may also be the trigger of a metabolic disorder decompensation [6], which ultimately was the case in our patient. A comprehensive clinical history, review of toxic and drug consumption, and a thorough laboratory investigation, including complete blood count, electrolyte evaluation, liver enzymes, serum bilirubin, coagulation studies, arterial blood gas, lactate, ammonia levels, CSF studies, and urine and blood screening (particularly for inborn errors of metabolism), are essential.

The differential diagnosis for AE is broad, including drug side effects, encephalitis, vascular diseases, and or metabolic disorders. Confirmation of brain edema through cranial CT/MRI, as well as EEG, which may show abnormal patterns such as diffuse, high voltage slow wave, electrical storm and absence of spindle waves, aids in the diagnosis. Syndromic diagnosis of AE may be necessary for prompt intensive care initiation [4].

Certain features, such as the absence of precursory episodes, mild respiratory disturbance, rapid clinical progression (with neurological fluctuation), absence of focalized neurological signs, and a history of familial inborn errors of metabolism may point towards AE caused by a metabolic disorder. Despite the importance of classifying AE into its multiple syndromes, treatment should not be delayed in pursuit of a final diagnosis, as this delay may result in a disastrous prognosis. In fact, our patient was previously healthy, with no familial history of inborn errors of metabolism, and showed a rapid progression of disease, similar to her sister, who did not survive.

The treatment of severe AE involves managing seizures (if present), pediatric advanced life support, and admission to a PICU for systemic management [4]. Actually, patients present with mostly central nervous system (CNS) disorders, which may guarantee them observation and monitorization of the level of consciousness (GCS) and brainstem reflexes, such as light, corneal and oculocephalic reflexes. ICP should be monitored if there is suspicion of ICH. In our case, the patient underwent surgical craniectomy due to severe ICH refractory to medical treatment, and empirical treatment with corticosteroids, tocilizumab, and endovenous immunoglobulin was administered, as it is the recommended treatment in other etiologies of AE such as acute necrotizing encephalopathy or AE caused by cytokine storm [4,7]. Thiamine supplementation was also empirically provided. Even with these efforts, the patient continued to show increased ICP for more than 10 days, with severe and challenging episodes of ICH.

After an initially inconclusive investigation, our patient received a definitive diagnosis of MSUD when exome sequencing revealed a pathogenic variant in homozygosity consistent with the condition. In Portugal, since 2005, MSUD is universally screened as part of the newborn metabolic screening program, but false-negative results, as seen in our patient and her sister, can occur, particularly in non-classical forms [8].

MSUD can manifest clinically at various life stages. The classical form typically appears in the newborn period, characterized by very low residual enzyme activity (less than 3%). The intermittent form, which is the second most common, presents with a higher residual enzyme activity and patients may exhibit normal growth and development. However, affected individuals often experience ketoacidosis during catabolic stress episodes. The intermediate form, a rare disorder, can present at any age, with the residual enzyme activity ranging from 3% to 30%. Thiamine-responsive MSUD, also rare, mirrors the intermediate form, with thiamine supplementation extending the enzyme's half-life. The E3-deficient form emerges in the newborn period, involving deficiencies in both BCKDC and the pyruvate and alpha-ketoglutarate dehydrogenase complexes [9].

While most cases of MSUD are diagnosed in the newborn period, non-classical forms may be identified later in life. Cases reported in the literature predominantly involve patients diagnosed during the onset of metabolic decompensations or during investigations into psychomotor development alterations, mental status impairment, or psychotic events [10,11].

Management of MSUD entails dietary therapy to support normal growth and development, and aggressive intervention during acute metabolic decompensation episodes. This includes high doses of glucose and specific amino acid formula, to redirect protein catabolism towards synthesis, and hemodiafiltration (if necessary), thus preventing short- and long-term sequelae [5].

Both our patient and her elder sister experienced simultaneous severe decompensation of an undiagnosed MSUD in their teenage years during an influenza infection. This led to the sister's death and to our patient’s prolonged complicated hospitalization, resulting in significant morbidity. Thus, this case, with a rare presentation of this uncommon disease, highlights the complexity of the already challenging diagnostic process.

## Conclusions

In summary, the clinical spectrum of AE is broad, encompassing a range of presentations from subtle to life-threatening conditions in severe cases. Timely diagnosis and classification within the diverse syndromes constituting this pathology are crucial due to their treatment implications. Importantly, therapeutic interventions, particularly in severe cases, should not be delayed, as such delays can adversely impact patient prognosis.

MSUD, like other inborn errors of metabolism, can contribute to the etiology of AE. While MSUD is typically screened during the newborn period, certain forms may only manifest later in life. A comprehensive understanding of the disease, including its diagnosis and treatment modalities, is imperative when managing a patient with AE to prevent misdiagnosis. Normal outcomes can be achieved by monitoring the disease, providing dietary support, and appropriately managing metabolic decompensations. Genetic familial counseling is advisable.
